# Seismic wave simulation using a 3D printed model of the Los Angeles Basin

**DOI:** 10.1038/s41598-022-08732-w

**Published:** 2022-03-17

**Authors:** Sunyoung Park, Changsoo Shin, Younglib Kim, Robert W. Clayton

**Affiliations:** 1 Department of the Geophysical Sciences, The University of Chicago, Chicago, IL 60637 USA; 2 Department of Energy Resources Engineering, Seoul National University, Seoul, 08826 South Korea; 3 Korea Advanced Machinery Inc., Seoul, 08502 South Korea; 4 Division of Geological and Planetary Sciences, California Institute of Technology, Pasadena, CA 91125 USA

**Keywords:** Natural hazards, Geophysics, Seismology, Civil engineering, Design, synthesis and processing

## Abstract

Studying seismic wave propagation through complex media is crucial to numerous aspects of geophysics and engineering including seismic hazard assessment. In particular, small-scale structure such as sedimentary basins and their edges can have significant effects on high-frequency earthquake ground motion, which is the main cause for the damage to buildings and infrastructure. However, such structural effects are poorly understood due to limitations in numerical and analytical methods. To overcome this challenge, for the first time, we utilize the 3D printing technique to build a scaled-down physical representation of geological structure and perform lab-scale seismic experiments on it. Specifically, a physical model based on the Los Angeles Basin is printed and used as synthetic medium to propagate ultrasonic waves, to mimic seismic wave propagation from local earthquakes. Our results show clear body and surface waves recorded at expected time and locations, as well as waves that are scattered from the basin edges. We find that high-frequency energies are significantly reduced at the basin, which is at odds with the conventional view of basins as ground motion amplifiers. This novel waveform modeling approach with 3D printed Earth models is largely automated and provides an effective means to tackle geophysical problems of significance.

Understanding the seismic wave propagation in complex media—with small-scale heterogeneities, rough topography and interfaces, anisotropy, or pore fluids—is crucial to various aspects of geophysics such as earthquake ground motion prediction, induced seismicity, and energy exploration. Some of these problems are difficult to address using numerical approaches not only due to constraints in computing resources, but also due to inaccuracies in forward models, assumptions and approximations that are being imposed. Alternatively, the challenges faced by the numerical approaches can be overcome by performing laboratory-scale seismic experiments on physical models. Physical-model based approaches have been used in exploration geophysics^1–3^ such that the seismic experiments mimic land and marine seismic surveys. However, these models are created by cutting and carving materials and combining them, and thus, have been mostly limited to representing simplistic geometries or coarse structures, e.g., a rectangular or cylindrical block embedded in a larger block. The models often represent only up to a few material properties that are predetermined by the materials of choice; for example, a model may be composed of 3 domains, each domain being plastic, acrylic, and aluminum. It is still possible to put together a relatively complex model, but it takes considerable effort, and it is difficult to replicate the model.

In this work, we take advantage of 3D printing techniques to create physical models for lab-based seismic experiments. Applications of the 3D printing techniques in earth sciences have been increasing^4,5^, mostly focused on visualization and education with some studies on hydromechanics and rock mechanics^6–11^. We demonstrate that the 3D printing opens up unprecedented opportunities for seismology in utilizing the physical model based seismic experiments. This is enabled by using the 3D printing techniques to synthetize physical models with desired structure and complexities, which is not possible in natural rock samples or simplistic physical models previously considered.

Here, we specifically design the 3D printed model and the seismic experiments for studying earthquake ground motion in the presence of sedimentary basins. As most populated cities and areas in the world are located at prominent basins, it is imperative to understand the effect of basins on the ground shaking. Observations have shown that basins amplify the ground motions in general^12–14^, and there have been numerous efforts to understand the amplification^15–20^. However, it remains difficult for the numerical approaches to study the effect of basins that are shallow, have irregular interfaces and edges, or multiple basins that are connected to each other. Incorporating these detailed structures and simulating seismic wave propagation, particularly, at high frequencies, have been challenging. To tackle these issues, we build a physical model containing basin structures using 3D printing and simulate the ground motion with the lab-generated quakes.

Therefore, the main purposes of this paper are (1) to introduce the new experimental approach using 3D printing and demonstrate its effectiveness and (2) to use this approach for simulating ground motions and examining the effect of sedimentary basins.

## 3D Printing of the Los Angeles Basin

**Figure 1:**
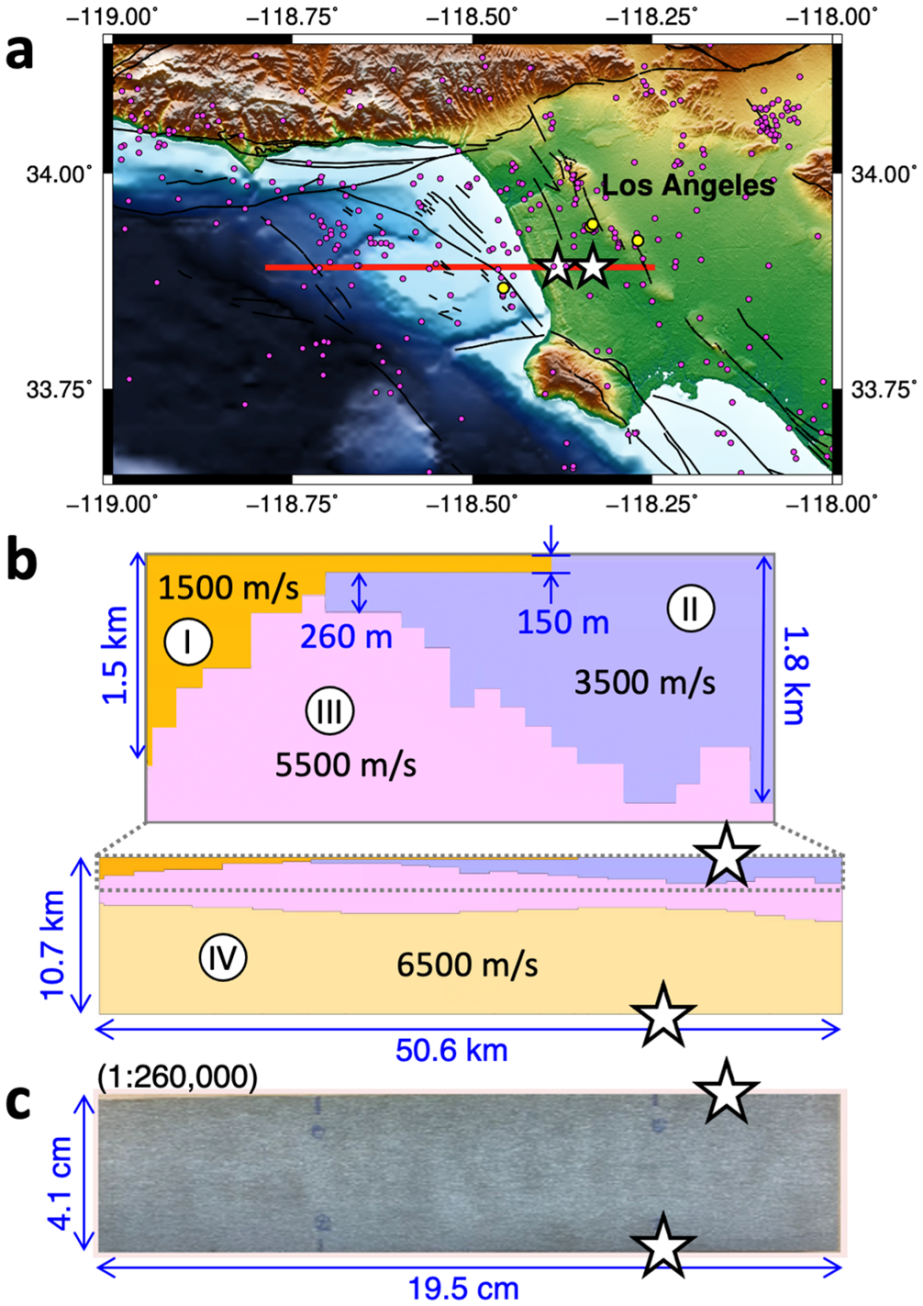
Study area, the velocity structure and the 3D printed model. (**a**) Topographic map of the Los Angeles area around the cross section considered in this study (solid red line). Locations corresponding to the two lab-generated quakes (white stars), local seismicity (magenta dots) with events noted in the text (yellow dots), and local faults (solid black lines) are shown. (**b**) A simplified velocity model of the 50.6-km long and 10.7-km deep cross-sectional area (corresponding to solid red line in (**a**)) plotted with the locations of the two lab-generated quakes (white stars) with a zoomed-in view of the shallow portion on top. The four domains are denoted with roman numerals with corresponding seismic P-wave speeds. (**c**) 3D printed version of (**b**) with the size of about 19.5 cm in length and 4.1 cm in height. The white stars are the same as ones in (**b**).

Our physical model example is based on the Los Angeles Basin, one of the most seismically active regions with large population, where understanding the structural effect on ground shaking is crucial to seismic hazard assessment (Fig. 1a). The model is derived from a 2D cross-section of a seismic velocity model^21^ that is 50.6-km long and 10.7-km deep. The model consists of four domains (I)–(IV), with compressional wave speeds of 1500, 3500, 5500, and 6500 m/s, simplified from the original velocity model (Fig. 1b). Note that the domain (I) of 1500 m/s corresponds to the ocean but is printed solid in this study and considered as a basin with the lowest seismic wave speed among the whole cross-sectional area. The two domains (I) and (II) can be regarded as sedimentary basins that are juxtaposed in the middle section of the cross-sectional area. The interface between the two basins and the domain (III) as well as that between the domains (III) and (IV) have variable topography along the cross section. The basins of (I) and (II) are 1.5- and 1.8-km deep near the west and east ends of the cross section and are as thin as 150 and 260 m at their edges, respectively. These basin depths are comparable to or significantly shorter than the wavelengths at 1 Hz, i.e., 1.5 and 3.5 km, for the domains (I) and (II). Such shallow and laterally varying basin structure has been recognized as a numerically challenging problem to study^20,22^ and its effect on ground motion is poorly understood.

To print the physical model composed of four domains with distinct material properties and irregular shapes, we use the metal 3D printing (see Methods for details). The simulated cross-section is 50.6-km long and 10.7-km deep and is printed into a 19.5 cm by 4.1 cm model, i.e., at about 1:260,000 ratio (Fig. 1c). The width of the model is about 10 mm.

## Seismic experiments on the 3D printed model

**Figure 2:**
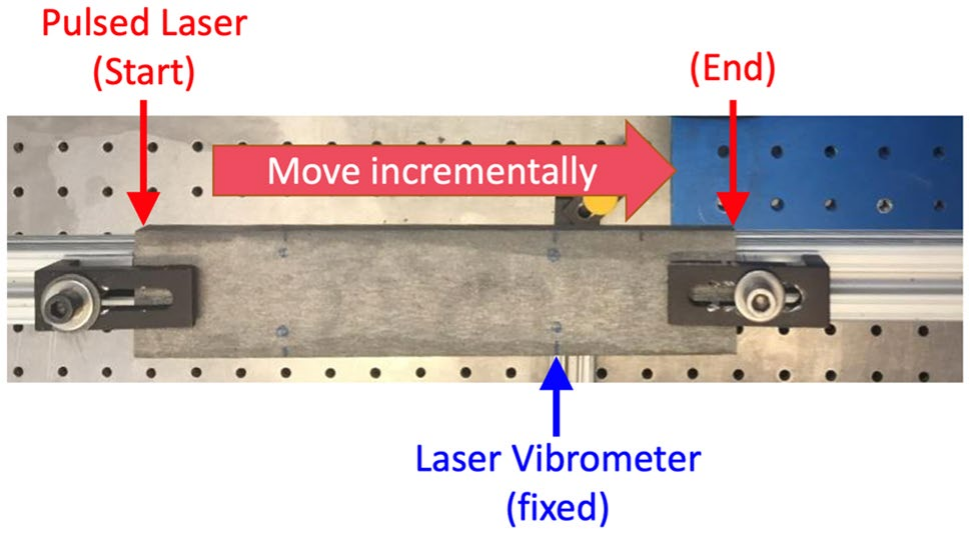
Setup for the seismic experiments on the 3D printed model. Thin arrows show the locations of the laser source (red) and receiver (blue). Thick red arrow shows the direction at which the laser is scanned along the top side of the model.

We use the 3D printed basin model to perform seismic experiments to investigate the effect of basin structure on ground motions. The ground motion is recorded along the top side of the model, from two separate source locations: one on the top surface and the other on the bottom surface, each located at about 3.1 and 5.0 cm from the right edge of the model, respectively (Fig. 1). Their corresponding geographical locations are close to active faults such as the Newport-Inglewood and Palos Verdes fault zones and numerous local earthquakes. Some of the notable earthquakes include 1988 M4.0 event near Manhattan Beach, 2001 M4.0 event near Willowbrook, and a sequence of events near Lennox such as 2009 M4.7 event and the recent 2021 M4.0 event. For the lab experiments, we utilize pulsed lasers as seismic sources and the laser doppler vibrometers as receivers (Fig. 2). In practice, we swap the locations of the source and receivers, i.e., fixing the vibrometer measurement to the source location while applying pulsed laser at the receiver location based on the reciprocity principle^23^. This allows avoiding potential degradation of the physical model from repeatedly hitting the source area with the pulsed laser.

The choice and set up of the source-receiver system are important in achieving the right scaling for the small-scale seismic experiments. Since the printed basin model has seismic wave speeds identical to the actual values while its size is reduced by the ratio of 1:260,000, we need to downscale the wavelengths at the same ratio in order to consider the equivalent wave propagation problem at the scale of Los Angeles basin. This can be achieved by simulating the seismic waves with frequencies that are 260,000 times higher than actual frequencies. The usual frequency range, e.g., 0.1–10 Hz, considered for the ground motion induced by local earthquakes corresponds to 0.026–2.6 MHz for the physical model, and seismic waves at such megahertz level of frequencies can be obtained by the pulsed laser and the laser vibrometer (see Methods for the details). The equivalence of the wave propagation problems in the actual and the laboratory scales can also be understood as the equivalence of the wave equations in both time and frequency domains:

$$\frac{{\partial^{2} u}}{{\partial t^{2} }} = c^{2} \frac{{\partial^{2} u}}{{\partial x^{2} }} \Leftrightarrow \frac{{\partial^{2} u}}{{\partial \left( {t/\alpha } \right)^{2} }} = c^{2} \frac{{\partial^{2} u}}{{\partial \left( {x/\alpha } \right)^{2} }}, and - {\upomega }^{2} {\text{U}} = {\text{c}}^{2} \frac{{\partial^{2} {\text{U}}}}{{\partial {\text{x}}^{2} }} \Leftrightarrow - \left( {{{\upalpha \upomega }}} \right)^{2} {\text{U}} = {\text{c}}^{2} \frac{{\partial^{2} {\text{U}}}}{{\partial \left( {{\text{x}}/{\upalpha }} \right)^{2} }},$$

where $u$ is the wavefield, ${\text{U }}$ is its Fourier transform, $x$, $t,{\text{ and }}\omega$ are space, time, and angular frequency, respectively, in the original scale, and $\alpha$ is a scaling factor, corresponding to 260,000 in this study.

**Figure 3:**
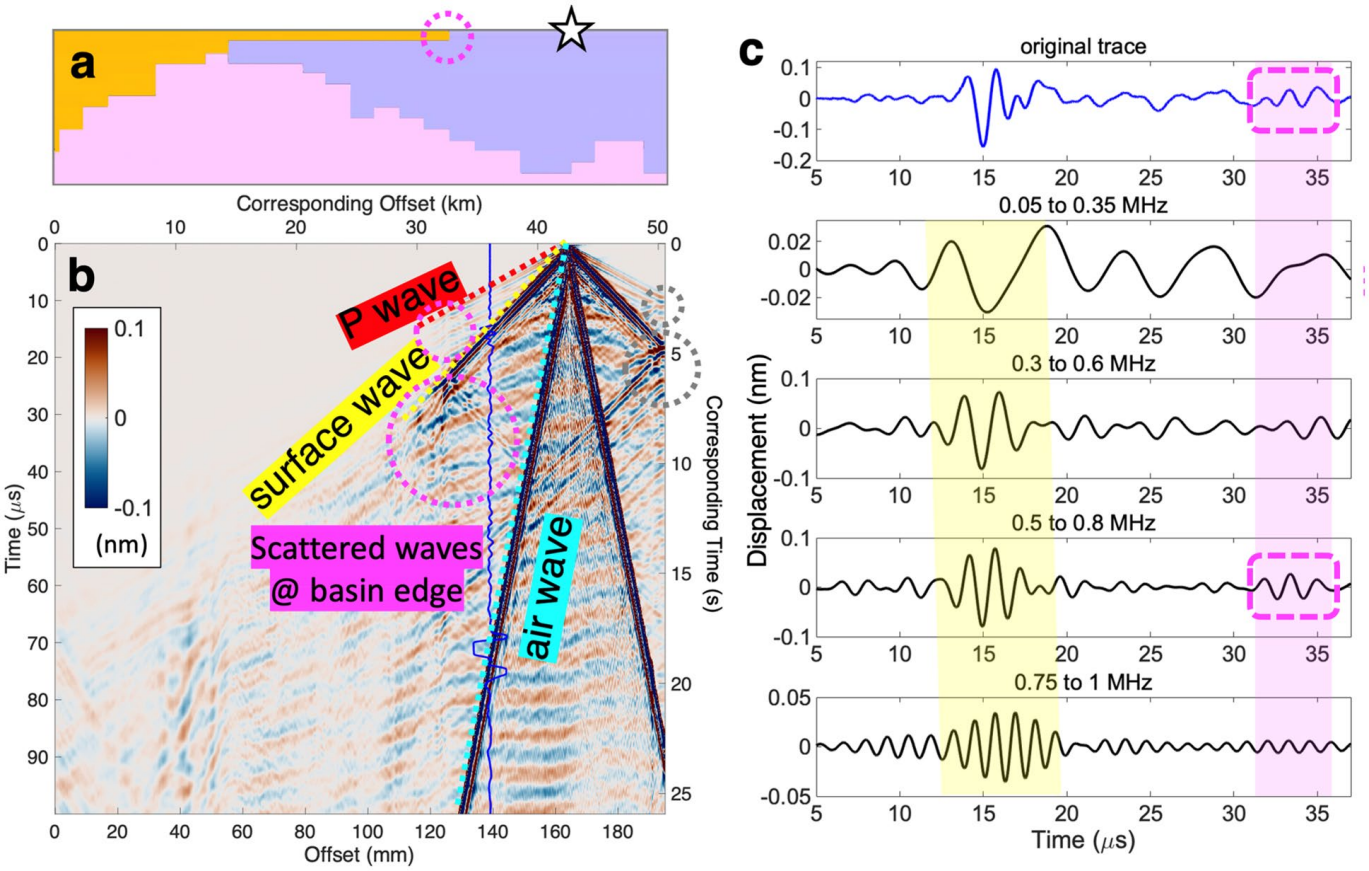
Seismic data obtained from the surface source experiments. (**a**) Top part of the velocity model shown in Fig. 1b with the source location (white star). The edge of the basin that is responsible for the scattered waves shown in (**b**) is highlighted with a dashed magenta circle. (**b**) Record section of the displacement time series shown in color at each point on the top surface with its offset (horizontal axis) measured from the left, for the surface source shown in (**a**). Additional axes for corresponding time (right) and offset (top) in the actual scale are presented. One of the timeseries is plotted with a solid blue line, a part of which is shown in the top panel of (**c**). P, surface, and air waves are marked with dashed red, yellow, and cyan lines, respectively. Waves scattered at the right edge of the model are highlighted with dashed gray circles. Those scattered at the basin edge shown with the dashed magenta circle in (**a**) are also highlighted with dashed magenta circles. An unlabeled version of the record section can be found in Supplementary Fig. S1. (**c**) Displacement recording (top) at an offset of about 140 mm corresponding to the blue trace in (**b**) is plotted on the top panel. The original recording is filtered at low to high frequency bands and each of them is plotted in the following panels with the corresponding frequency band shown on the top of each panel. Recordings capture two surface wave trains: one coming directly from the source (between 10 and 20 μs; yellow shading highlighting the dispersion) and the other that is scattered from the basin (after 30 μs; magenta shading). Dashed magenta boxes encapsulate the two similar waveforms of the scattered wave in the original trace and the filtered trace with frequencies from 0.5 to 0.8 MHz.

To demonstrate that our seismic experiments with the 3D printed model produces the expected seismic phases as in the seismic wave propagation on Earth, we first discuss the relatively simple case of the surface source (Fig. 3a). This case resembles active-source seismic experiments or other shallow source processes in the study area. The surface source efficiently generates high-amplitude surface waves, which makes it also suitable for investigating how surface waves propagate and interact with the basin structure in general, for local or distant earthquakes. We measure the displacement at every 200 µm along the surface, which corresponds to about 52-m sampling in the field. At each point, the displacement is recorded for 100 µs at the sampling rate of 50 MHz. The measurements are repeated 100 times and averaged to obtain a high-quality signal. The obtained seismograms can be translated into the original scale by simply stretching the time dimension by 260,000 times, and they become equivalent to about 26-s long recordings at a 192-Hz sampling rate (Fig. 3b). It is worth highlighting that the recording process is largely automated, and it takes about 1.4 h to obtain a record section (see Methods for the details).

Since both source and receivers are at the surface, different seismic phases with distinct traveling speeds appear with distinct slopes in the record section and can be easily identified (Fig. 3b). The first arriving energy is the P wave. It exhibits the slope corresponding to about 3500 m/s around the source location, which matches the wave speed of the domain (II). The high amplitude energy arriving afterwards with the slope corresponding to about 1700 m/s is the surface wave. Filtering this wave into different frequency bands exhibits the dispersion phenomenon, where low frequency energy arrives earlier than the high frequency energy (Fig. 3c). The dispersion is apparent in the frequency range of 0.05–1 MHz, which corresponds to about 0.19–3.85 Hz in the actual domain. Note that the distinct energy arriving last with the slope of about 350 m/s is the air wave (See Supplementary Information for details). We do not observe clear S waves since only vertical motions are recorded and discussed in this work.

In addition to the direct waves, there are reflected waves that stand out. Both P and surface waves show clear reflections at the free surface on the right-side boundary of the physical model (Fig. 3b). The reflected waves are characterized by the slopes that are the same with those of the direct waves. Similarly, the free surface at the bottom-side boundary of the model would generate reflected P waves, but their amplitudes are too small to be clearly observed in the record section. Such reflections do not exist in the wave propagation in actual continuous medium, and one should be careful when interpreting the whole record section. Note that these reflections are clearly separated from the main phases that we discuss in this study.

Interestingly, we observe similar reflections at the edge of the domain (I) characterized by the lowest wave speeds among all parts of the model (Fig. 3b). There are reflections of both P and surface waves, and the amplitude of the reflected surface wave is significant enough to be observed clearly in individual traces (Fig. 3c). We find that there is a marked difference in the frequency contents of the direct and reflected surface waves. While the direct wave has a relatively broad frequency content from 0.05 to 1 MHz, with 0.3 to 0.8 MHz being dominant, the reflected wave is mainly at 0.5 MHz and higher. Majority of the reflected wave is explained by the energy between 0.5 and 0.8 MHz, considering its waveform and the amplitude shown in the original trace is almost identical to what appears in the frequency band of 0.5–0.8 MHz. In the actual scale, 0.5 MHz translates to about 1.92 Hz, which corresponds to the wavelengths of about 780 and 1820 m for the domains (I) and (II) of 1500 and 3500 m/s, respectively. Thus, the results indicate that the shallow basin that is 150-m deep at the edges effectively reflects seismic energy of 1.92 Hz and higher, wavelengths of which are more than 5 times longer than the basin depth.

**Figure 4:**
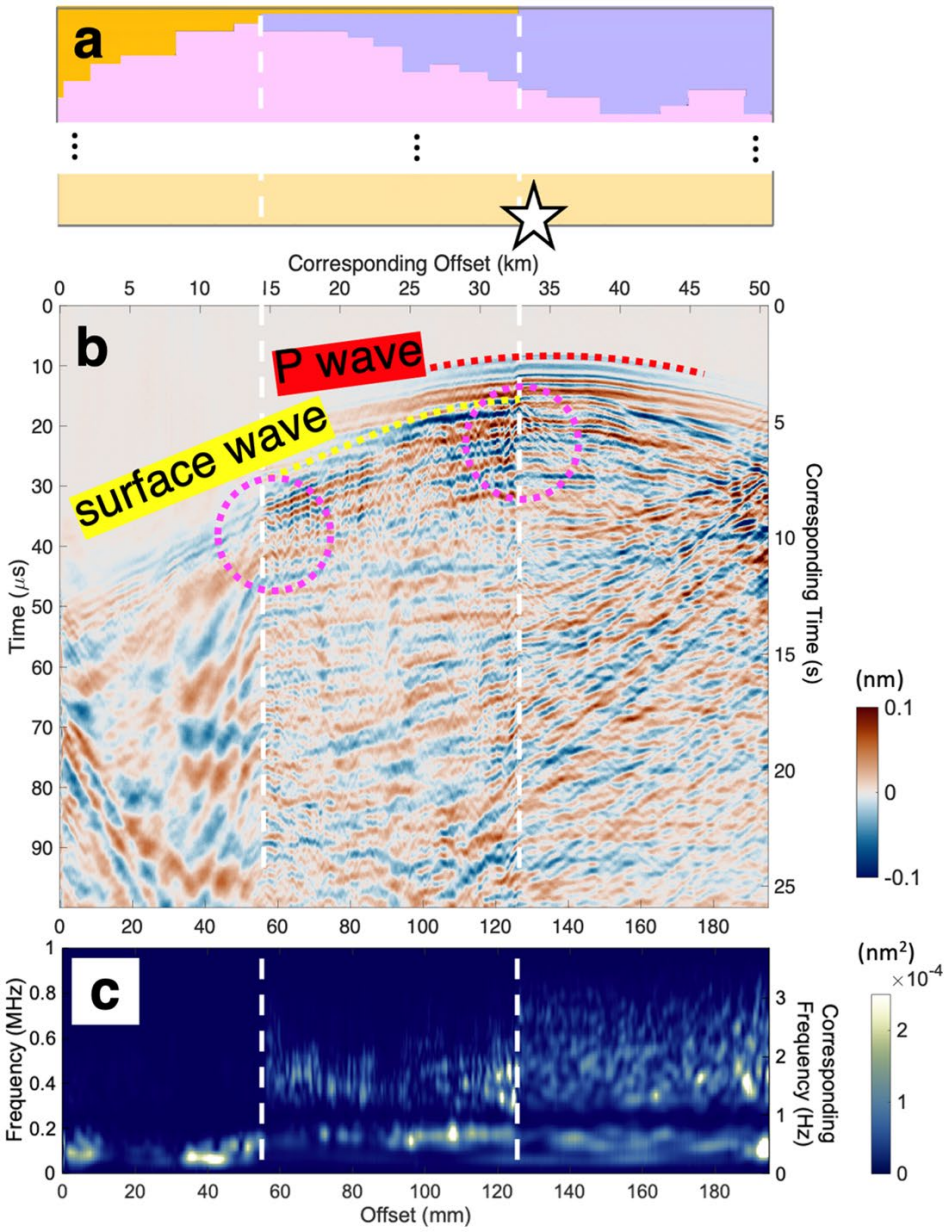
Seismic data obtained from the experiments with the source at depth. (**a**) The velocity model shown in Fig. 1b with the source location (white star). The two edges of the basins are marked with the dashed white lines. (**b**) Same as Fig. 3b except for the record section corresponds to the deep source shown in (**a**). P and surface waves are marked with dashed red and yellow lines, respectively. Waves scattered at the two basin edges (marked by dashed white lines) are highlighted with dashed magenta circles. An unlabeled version of the record section can be found in Supplementary Fig. S2. (**c**) Spectrum of each displacement time series in (**b**) at each offset shown in color. Two vertical axes are presented: frequencies in the experiments on the left and their corresponding frequencies in the actual scale.

After confirming the observations of the body and surface waves that are consistent with the input velocity model from the surface source experiments, we have performed a similar set of experiments with the source located at the bottom, corresponding to 10.7 km in depth (Fig. 4). Since the source is at depth, the traveltimes of the first-arriving P wave exhibit curvature, followed by surface waves with relatively large amplitudes (Fig. 4b). Similar to the previous case with the surface source, the surface waves are scattered at the edges of the two basins (domains I and II). The scattering is observed the most prominently for the direct surface waves, but it is occurring throughout the record section, i.e., for the later arriving waves as well. Such scattering at the basin edges results in the striking difference in frequency contents in the seismic records. The record section can be divided into three groups, marked by the two edges of the two basins. This becomes obvious when spectrum of each seismic record is examined (Fig. 4c). There are abrupt changes in the frequency contents at the basin edges, where high frequency energies above about 0.55 and 0.15 MHz are subsequently lost as you go from the right to the left. In the actual scale of the Los Angeles Basin, these frequencies correspond to about 2.1 and 0.6 Hz. These high frequency waves are mostly reflected back at the basin edges (Fig. 4b) and barely travel through the low-wave speed basin (domain I). Such diminution of high frequency energies in the prominent basin is at odds with the general understanding of ground motion amplification within basin structures, possibly because most studies explore relatively low-frequency responses. At frequencies below 0.6 Hz, on the other hand, the general understanding seems to hold considering that the energies are relatively large on top of the domain I compared to those of II.

We note that seismic attenuation, i.e., 1/Q, would also contribute to frequency-dependent reduction of the ground motion, but the effect is secondary. Sedimentary basins generally have a relatively high attenuation, or low Q^24–26^, resulting in gradual decrease in seismic energies with distance. This would lead to a progressive decline in high frequency contents both within and across basins, which cannot explain our observations of the instantaneous depletion of high-frequency energy at the edges of basins (Fig. 4c). There is no obvious sign of progressive decrease in frequency contents within each domain, suggesting that the effect of Q is not as significant. Moreover, the near-perfect reflection of the high-frequency waves (dashed magenta boxes in Fig. 3c) supports that the reflection due to the contrast in seismic wave speeds is the prominent cause of the high frequency reduction, rather than the attenuation. This is compatible with studies showing the basin effects that are primarily due to the contrast in elastic structure^19,20^.

## Conclusions

Our results demonstrate that the effect of basin structures in ground motions is highly dependent on the frequency contents, and basins can, in fact, reduce the high-frequency energies by scattering them away. We show such scattering effect is significant at the relatively long wavelengths that are 5 times or longer than the basin depth. Therefore, basins that are only fractions of the considered wavelengths must be taken into account when studying the ground motion.

This study introduces and validates the new seismic modeling approach using 3D printed Earth models. The modeling is highly effective since it is independent of the complexity of the structure, theoretical or numerical assumptions, and computational grids. Automation of the data acquisition process also makes the modeling largely hands-off. Therefore, this approach provides a powerful means of tackling geophysical problems of significance beyond the domain of earthquake ground motions.

## Methods

### Metal 3D printing of the physical model

Unlike other materials such as plastic and resins, metal can represent a medium as rigid as the Earth’s lower crust at standard laboratory conditions (1 atm., 25 °C). In this study, stainless steel powder (KS standard material STS316L) that has density of about 8 g/cm^3^ and the elastic moduli of about 200 GPa, is used. Printing is carried out by the Concept Laser M2 Cusing 3D Metal Printer which applies direct metal laser melting technique, i.e., creating components layer by layer by sintering metal powder with high-energy laser. Such metal printing technique allows us to represent material properties that are highly variable within a medium, which is a crucial component of creating physical models of geological structure. In this study, we achieve this by adjusting the scanning speed of the laser during the printing process, which effectively controls the density, and thus, the wave speed of the printed model. Based on the inverse relationship between the laser scanning speed and the compressional wave speed, the domains (I)–(IV) with compressional wave speeds of 1500, 3500, 5500, and 6500 m/s are printed with the scanning speeds of 2500, 1600, 800, and 400 mm/s, respectively, at a constant laser power of 180 W. Even though attenuation (1/Q) has not been measured in this study, it is noteworthy that we expect higher attenuation (lower Q) for the domains with lower wave speeds (and lower density) and vice versa. The thickness of each layer is 50 µm, which corresponds to 13 m in the actual scale at the 1:260,000 ratio.

### Experimental setup

The pulsed laser used in this study is the Lumibird CFR200, a Nd:YAG pulsed laser. The laser wavelength is 1064 nm, the pulse duration is 12 ns, and the energy per pulse is 20–30 mJ. For the laser vibrometer, the Sound & Bright Quartet interferometer is used. It has the wavelength of 532 nm, and the laser power of 500 mW. Its detection bandwidth is 9 MHz, and the detection spot size is about 50 µm. This laser-based source/receiver system provides high resolution and accurate measurements that are superior to what conventional piezoelectric transducers can provide^27,28^; (1) there are no coupling issues that transducers often suffer from, since laser system do not require a physical contact the model, and (2) the lasers can be focused to the tens of micrometers, providing the resolution remarkably superior to the transducers with the finite contact area of about 5 to 10 mm in diameter. For precise positioning of the sample and the source and receiver, Velmex 2-axis translation stage has been used. The motor-driven translation stage automates the incremental movement of the source or receiver location (Fig. 2) and makes the data acquisition process largely hand-off. The amount of time it takes to obtain a record section depends upon the number of measurements at each point, the number of the measurement points, and the repetition rates of the pulsed laser. In this study, it takes about 1 h and 20 min to obtain a record section with about 975 measurement points and 100 measurements per point at 20 Hz repetition rate. We repeat the measurement for 100 times per point to achieve exceptionally high signal-to-noise ratios over 30 dB, but one can reduce the number of repetition and still obtain a sufficiently high-quality data within a relatively short time span. Even though this study is limited to the vertical component, there are experimental setups available for applying horizontal (shear) sources and measuring horizontal motions.

## Supplementary Information

Supplementary Information.

## Data Availability

Seismic data obtained in this study is available at an online data repository^29^ (10.5281/zenodo.6350691).
