# Supplementary material for: Seismic wave simulation using a 3D printed model of the Los Angeles Basin
Source: Sci Rep. 2022 Mar 17;12:4613. doi: 10.1038/s41598-022-08732-w (PMC8931089; doi:10.1038/s41598-022-08732-w)
Supplement: Supplementary file 1 — Supplementary Information. [file 41598_2022_8732_MOESM1_ESM.docx]

Supplementary Information

**Air Wave.** The air wave exhibits the slope corresponding to about 350 m/s, matching the sound wave speed in the air (Fig. 3b). Since the wave travels outside the physical model, there is no reflection of energy at the boundaries of the model that are observed for P and surface waves. The high amplitude recordings of the air wave result from the explosive characteristics of the source and the sensitivity of the laser vibrometer to the motion in the air. Infrasound recordings of surface sources such as volcanic or nuclear explosions are analogous to the recorded air waves. Note that the air wave is recorded effectively only when both source and receiver are on the same surface. Therefore, they do not appear in the deep source experiments (Fig. 4b).


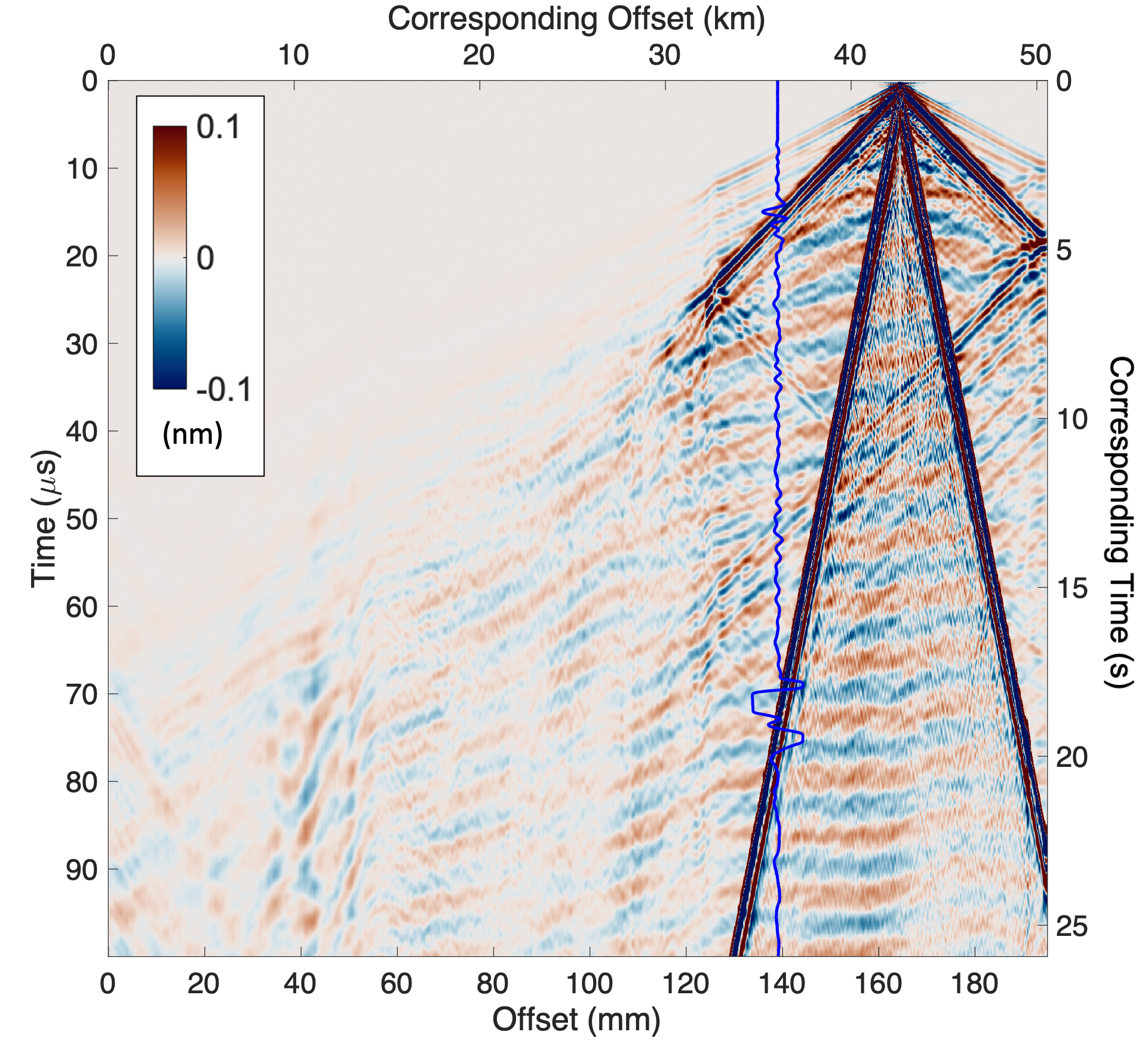


Figure S1. Seismic data obtained from the surface source experiments.

An unlabeled version of the Fig. 3b.


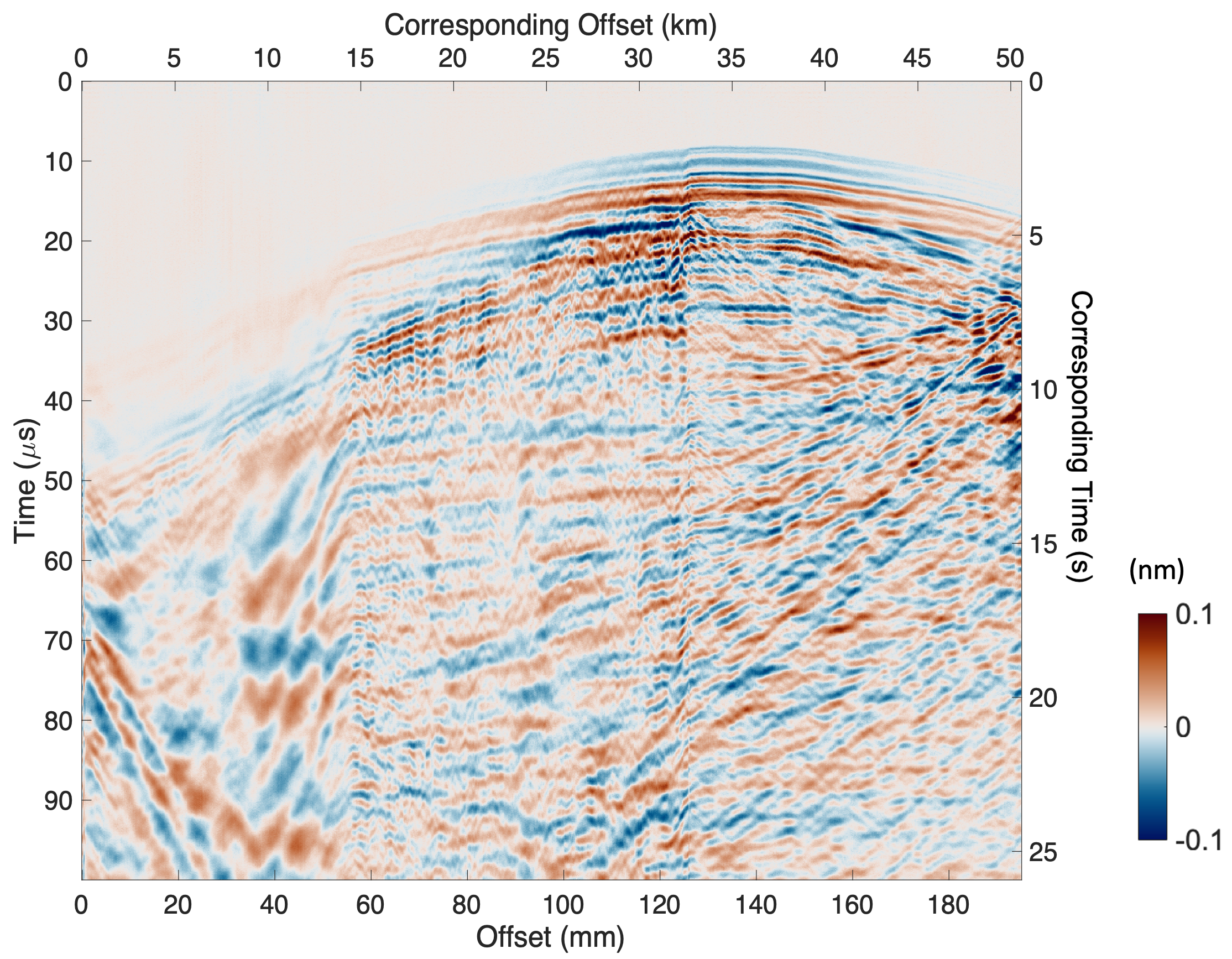


Figure S2. Seismic data obtained from the experiments with the source at depth.

An unlabeled version of the Fig. 4b.
